# Ionomer-Liquid Electrolyte Hybrid Ionic Conductor for High Cycling Stability of Lithium Metal Electrodes

**DOI:** 10.1038/srep14458

**Published:** 2015-09-28

**Authors:** Jongchan Song, Hongkyung Lee, Min-Ju Choo, Jung-Ki Park, Hee-Tak Kim

**Affiliations:** 1 Department of Chemical and Biomolecular Engineering, Korea Advanced Institute of Science and Technology (KAIST), 291 Daehak-ro, Yuseong-gu, Daejeon 305-338, South Korea

## Abstract

The inhomogeneous Li electrodeposition of lithium metal electrode has been a major impediment to the realization of rechargeable lithium metal batteries. Although single ion conducting ionomers can induce more homogeneous Li electrodeposition by preventing Li^+^ depletion at Li surface, currently available materials do not allow room-temperature operation due to their low room temperature conductivities. In the paper, we report that a highly conductive ionomer/liquid electrolyte hybrid layer tightly laminated on Li metal electrode can realize stable Li electrodeposition at high current densities up to 10 mA cm^−2^ and permit room-temperature operation of corresponding Li metal batteries with low polarizations. The hybrid layer is fabricated by laminating few micron-thick Nafion layer on Li metal electrode followed by soaking 1 M LiPF_6_ EC/DEC (1/1) electrolyte. The Li/Li symmetric cell with the hybrid layer stably operates at a high current density of 10 mA cm^−2^ for more than 2000 h, which corresponds to more than five-fold enhancement compared with bare Li metal electrode. Also, the prototype Li/LiCoO_2_ battery with the hybrid layer offers cycling stability more than 350 cycles. These results demonstrate that the hybrid strategy successfully combines the advantages of bi-ionic liquid electrolyte (fast Li^+^ transport) and single ionic ionomer (prevention of Li^+^ depletion).

High energy density levels and long lifetimes of secondary batteries have been ceaselessly pursued with the rapid evolution of electric vehicles and state-of-the-art mobile devices. Rechargeable lithium ion batteries (LIBs) have been widely used in these applications due to their high operation voltage, capacity, and acceptable durability; however, they have reached limits in their performance due to the inherent low specific capacities of the graphitic carbon (LiC_6_) used for the anode and of the transition metal oxides (i.e., LiCoO_2_) used for the cathode. In this regard, rechargeable lithium metal batteries (LMBs), in which graphite at the anode is replaced with high-capacity lithium (Li) metal (3,860 mAh g^−1^) have attracted attention given the expectation of their capacity to mitigate the aforementioned shortcomings of LIBs^1^.

The major challenge in the development of LMBs is to prevent inhomogeneous Li electrodeposition on the Li metal surface during repeated charge/discharge cycling, which results in dendritic/mossy Li growth on the Li metal electrode. The growth of the dendritic/mossy Li accelerates electrolyte decomposition reactions and results in low coulombic efficiencies of the cell. Also, the Li dendrite can lead to sudden cell failure owing to short-circuiting^2^. Researchers have devised and tested numerous strategies to realize uniform and reversible Li deposition, including mechanical dendrite blocking by solid ceramic or polymer electrolytes^3^,^4^,^5^,^6^,^7^, the *in-situ* modulation of a solid-electrolyte interface (SEI) by electrolyte additives^8^,^9^,^10^,^11^,^12^,^13^,^14^,^15^, *ex-situ* Li metal surface coatings^16^,^17^,^18^,^19^,^20^,^21^,^22^, and electrostatic shielding with a self-healing agent^23^,^24^. Although these approaches have shown promising enhancements, attaining high cycling efficiency at the high current densities (~10 mA cm^−2^) required for the practical design of these batteries remains challenging.

On the other hand, theoretical studies of the formation of Li dendrite have provided a deeper understanding of the mechanism and new insight into realizing homogeneous Li electrodeposition. Brissot *et al.*^25^ described the anionic and Li^+^ concentration gradients near the Li metal surface and the consequent local space charge based on a pacesetting study by Chazalviel^26^, indicating that Li dendrite formation starts after the Sand time (τ), at which the ion concentration drops to zero at the Li surface. According to this model, τ is dependent on the anion transference number (t_a_), as given in equation (1),


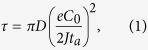


where e, D, C_o_, and J denote the electric charge, ambipolar diffusion coefficient, initial concentration, and applied current density, respectively. In addition, t_a_ is correlated the with transference number of Li^+^ 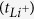 and the mobility of the anion (*μ*_*a*_) and Li^+^ 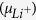, as given in equation (2) below.


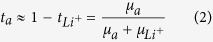


An important prediction from the theory is, when 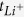 approaches unity, Li dendrite formation can be infinitely retarded owing to the constant Li^+^ concentration at the Li interface caused by immobilized anions. A recent model by Tikekar *et al.* also suggested that a single-ion Li^+^ conducting electrolyte with an immobilized anion would result in the stable electrodeposition of all metals, which was further experimentally validated by tethering anions to particles^27^,^28^,^29^. Several single-ion conducting materials have long been reported as an electrolyte for LMBs^30^,^31^,^32^,^33^,^34^,^35^,^36^,^37^,^38^,^39^. However, their poor chemical/electrochemical stability levels or low room-temperature ionic conductivity values have limited their practical application in LMBs thus far. Very recently, Nafion, a typical perfluorinated ionomer with a mechanically stable polytetrafluoroethylene backbone and a perfluorovinylether side chain with a lithium sulfonate end group, was revisited to improve the cycling stability of the Li metal electrode^39^; a Li/Li symmetric cell employing a Nafion polymer electrolyte membrane (NPEM) plasticized by EC/DEC exhibited significantly improved cycling stability. However, its test current densities were as low as 0.065 mA cm^−2^, the overvoltages were larger than 100 mV, and the interfacial resistance was approximately ~270 Ω cm^2^, thus preventing a practical battery design.

In this paper, we propose a thin Nafion layer (NL) as a functional coating layer on a Li metal electrode coupled with a conventional bi-ionic liquid electrolyte (LE), which can significantly enhance the cycling stability of LMBs and permit its room-temperature operation. The key to the success of this approach is in three aspects: i) a thin layer of Nafion as a single-ion conductor is placed on the surface of the Li metal electrode to prevent Li^+^ depletion at the interface, ii) the bulk resistance of the LMB is minimized by reducing the thickness of the NL down to a few microns and incorporating a bi-ionic LE, iii) lamination of the NL and Li metal electrode provides a tight bonding and consequent uniform interface, preventing direct contact between the LE and the Li metal electrode. In particular, the strategy of hybridizing single-ion conducting NL and a bi-ionic LE allows rapid Li^+^ transport and stable Li electrodeposition, which is the unique feature of this study and is not practically possible using either a single-ion conductor or a bi-ionic LE. The physical basis of this approach is that ramified Li deposition is triggered by surface instability due to an electric field with a large interface, as suggested by Chazalviel^26^, and that the concentrated and bulk polarizations near the Li metal electrode should be lowered to prevent such a high interface electric field. For the hybrid approach, the Li^+^ concentration at the Li metal surface does not fall to zero owing to the presence of fixed sulfonate anions near the Li metal surface, although the use of a LE results in a 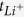 value lower than unity. In addition, the incorporated LE lowers the ohmic polarization of the battery and permits room-temperature battery operation at high current densities. As a proof of principle and to demonstrate the efficacy of the proposed approach, we comparatively investigated the Li electrodeposition/dissolution behaviours of Li/Li symmetric cells based on bare Li metal electrodes coupled with a LE (1 M LiPF_6_ in EC/DEC (1/1)), thin NLs coated onto Li metal electrodes coupled with the LE (NL/LE hybrid), and bare Li metal electrodes coupled with an EC/DEC-swollen NPEM (Fig. 1). Also, a Li/LiCoO_2_ prototype cell employing a NL-coated Li metal electrode was designed and assessed to demonstrate the practical applicability of this approach.

## Results and Discussion

**Figure 2:**
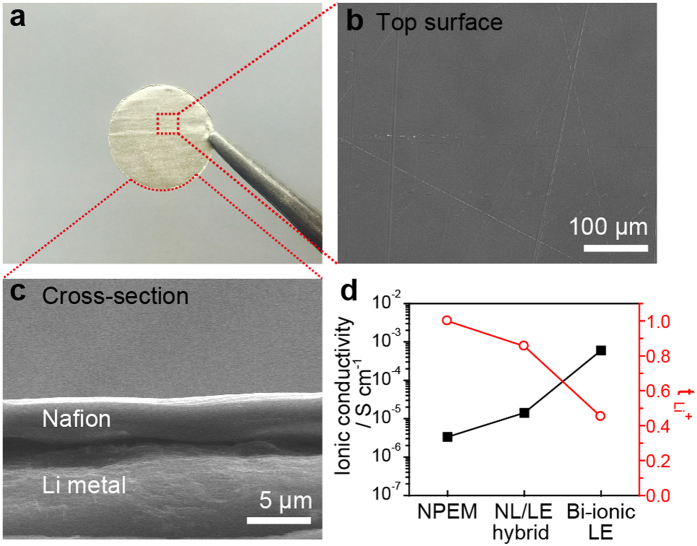
Morphology of the thin NL-coated Li metal electrode and electrochemical characterization. (**a**) digital photo and SEM images of the (**b**) top surface and (**c**) the cross-section of the pristine NL-coated Li metal electrode. (**d**) Comparison of the ionic conductivities and 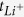 values of SUS/SUS and Li/Li symmetric cells with LE, the NL/LE hybrid, and NPEM, respectively.

The uniformity of the NL when formed on a Li metal electrode is crucial because any defects or thinner parts with lower resistance can trigger localized Li electrodeposition. Moreover, exposure of the Li metal electrode to a solvent which can cause surface passivation should be avoided. Hence, we utilized a decal transfer technique for the fabrication of the NL-coated Li metal electrode. In this case, a solvent-free, dried NL on fluorinated ethylene propylene (FEP) film was transferred to a Li metal electrode by applying pressure at room temperature. As a result, a dense, 4-μm-thick NL covering the entire surface of the Li metal electrode was fabricated without any defects such as cracks or holes, as shown in Fig. 2a–c. This room-temperature bonding process, known as pressure-sensitive cold lamination, is widely used in the bonding of various polymers and metals^40^. It should be noted that for easier decal transfer, protonated Nafion (H-Nafion), which is more flexible than Li-Nafion, was used but was transformed into Li-Nafion under galvanostatic cycling, as indicated by a FT–IR analysis (Supplementary Fig. S1).

The ionic conductivity and 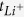 values for the LE, NL/LE hybrid, and NPEM are comparatively displayed in Fig. 2d. The ionic conductivity of the NL equilibrated with LE (1.42 × 10^−5^ S cm^−1^) was more than four times higher than that of the NPEM (3.33 × 10^−6^). Using the method devised by Bruce and Vincent^41^, the 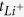 values were determined to be 0.451, 0.855, and 1.000 for the LE, NL/LE hybrid, and NPEM, respectively. More detailed experimental results and calculations are given in Supplementary Fig. S2. The 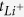 value of unity for the NPEM indicates single-ion conduction, which is in good agreement with the findings of previous works^42^,^43^. The higher 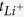 for the NL/LE hybrid compared to that for the LE demonstrates that the contribution of Nafion to the total Li^+^ conduction is significant for the NL/LE hybrid.

**Figure 3:**
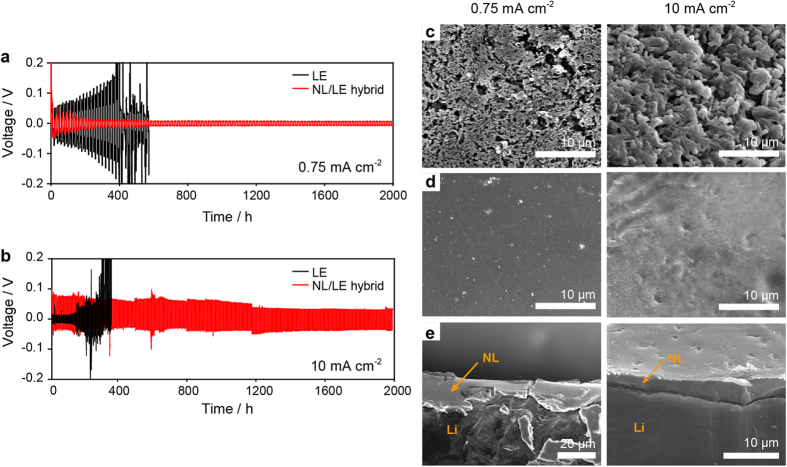
Comparison of the Li deposition/dissolution behavior between the LE and NL/LE hybrid cell. Galvanostatic cycling curves of the NL/LE hybrid (with the NL-coated Li metal electrode, shown in red) and the LE (with a bare Li metal electrode shown in black) (**a**) at a fixed current density of 0.75 mA cm^−2^, where each half-cycle life lasts for 10 h, and (**b**) at a fixed current density of 10 mA cm^−2^, where each half-cycle life lasts for 4 h. SEM images of (**c**) the surface of the bare Li after 200 h (10 cycle) at 0.75 mA cm^−2^ and after 80 h (10 cycle) at 10 mA cm^−2^, (**d**) the surface of the NL on the Li metal after cycling, and (**e**) the cross-section of the NL-coated Li metal electrodes after cycling.

Then, we investigated the Li^+^ electrodeposition behaviours of a Li/Li symmetric cell with the NL-coated Li metal electrode (NL/LE hybrid cell) and compared these findings with those of a cell with a bare Li electrode employing LE (LE cell). Two different galvanostatic cycling tests were performed; one consists of 10 h dissolution and 10 h deposition per cycle at a current density of 0.75 mA cm^−2^, and the other has a 4 h dissolution time and a 4 h deposition time per cycle at a current density of 10 mA cm^−2^. The operating conditions correspond to the dissolution and deposition of Li 3.7 μm and 19.7 μm thick, respectively. It should be noted that the NPEM swollen with EC/DEC did not operate due to its large polarization at these high current densities. A comparative investigation between the NL and NPEM at lower current densities will be presented later in this section. The results after galvanostatic cycling at 0.75 mA cm^−2^ are shown in Fig. 3a. For the LE cell, the overvoltages increased with cycling. Such an increase in the overvoltage is generally understood to result from electrolyte decomposition on the Li surface promoted by the increased Li surface roughness^44^ and successive accumulation of the resistive decomposition products. The LE cell showed a sudden polarization drop at the 20^th^ cycle, and its operation failed at the 30^th^ cycle, which is typical of short-circuiting behavior. In sharp contrast, the NL/EL hybrid cell exhibited lower polarization in comparison with the EL cell along with stable cycling for more than 100 cycles (2000 h). Even at a high current density of 10 mA cm^−2^, the NL/LE hybrid cell exhibited significantly improved cycling stability over the LE cell, as demonstrated in Fig. 3b. The LE cell became destabilized after the initial 19 cycles, and the cycling failed at 43 cycles (344 h). Although the overvoltages of the NL/LE hybrid cell were larger than those of the LE cell in early cycles, these were stably maintained for more than 250 cycles (2000 h) upon cycling.

The significantly improved cycling stability of the NL/LE hybrid cell suggests its higher reversibility under Li deposition/dissolution. To confirm this, the morphologies of the Li metal electrodes were investigated by SEM after 10 cycles at 0.75 and 10.0 mA cm^−2^. SEM images of the bare Li metal electrodes from the NE cells show the formation of a moss-like porous layer over entire electrode surface (Fig. 3c) for the two cycling conditions. For the NL/EL hybrid, the uniform and flat NLs were preserved (Fig. 3d). These types of porous Li layers were not formed at the NL/Li interfaces, and the NLs were found to be tightly adhered to the Li metal electrodes, as shown in the cross-sectional SEM image of the NL-coated Li metal electrode after cycling (Fig. 3e). The NL was not easily separated from the Li metal electrode, which made the direct observation of the Li metal electrode surface difficult. However, the Li metal surface observed under the partially peeled NL was highly uniform, as shown in Supplementary Fig. S3. Therefore, it can be concluded that the NL/LE hybrid design provides uniform Li deposition/dissolution even when 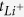 is less than unity. Due to the fixed anion and charge neutrality principle, the Li^+^ concentration at the Li metal interface should be higher or, at least, equal to that of the fixed anion, preventing Li^+^ depletion at the interface. On the other hand, the stabilized Li electrodeposition can be attributed to the mechanical suppression of dendritic Li growth by the NL^22^,^45^. However, the mechanical shear modulus of the NL/LE hybrid as measured by the nano-indentation method was lower than the critical value required for mechanical suppression, indicating that mechanical suppression is not the major mechanism stabilizing the Li (Supplementary Fig. S4).

**Figure 4:**
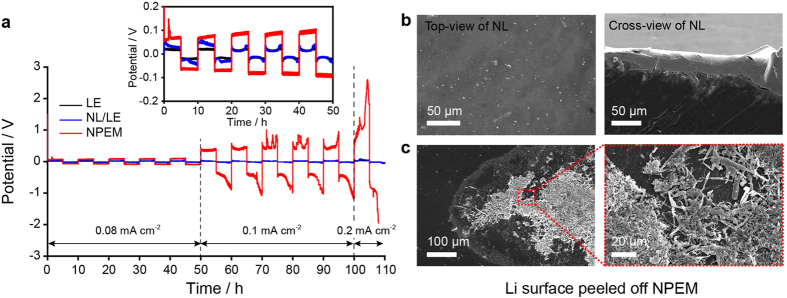
Comparison of Li deposition/dissolution between the NL/LE and NPEM cell. (**a**) galvanostatic cycling curves of Li/Li symmetric cells based on the LE, NL/LE, and NPEM at current densities of 0.08 to 0.2 mA cm^–2^, where each half-cycle life lasts for 5 h. (**b**) SEM images of the surface and cross-section of the NL-coated Li metal electrode of the NL/LE cell, and (**c**) the surface of the Li metal electrode of the NPEM cell after 20 cycles (100 h).

As demonstrated recently^39^, NPEM swollen with polar solvents can provide homogeneous Li electrodeposition at low current densities (0.065 mA cm^−2^ at room temperature) owing to the high 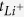 value in this case; however, the ohmic polarization was significantly high (>100 mV) due to the considerable thickness (50 μm) and low ionic conductivity. In comparison with the NPEM, the NL/LE hybrid has higher ionic conductivity but a lower 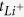 value. A question raised here is how important ohmic polarization in uniform Li electrodeposition is. In our approach, LE is introduced to lower the ohmic polarization at the expense of 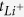. To address this question and prove the efficacy of the hybrid approach, we compared the cycling stability of Li/Li symmetric cells utilizing the NPEM and the NL/LE hybrid at low current densities. Upon galvanostatic cycling, the NPEM cell exhibited a large overvoltage of 0.1 V at a current density of 0.08 mA cm^−2^, which is approximately five times larger than those of the LE and NL/LE hybrid cell (inset of Fig. 4a). Moreover, the NPEM cell showed a gradual increase in its overvoltage as cycling proceeded, while the other cells exhibited nearly constant overvoltages. The difference in the overvoltage was manifested at subsequent cycling at higher current densities. The overvoltage of the NPEM cell increased to 0.4 V at 0.1 mA cm^−2^ and eventually reached an instrumental voltage limit at 0.2 mA cm^−2^ (Fig. 4a). After the initial ten cycles, the other set of the symmetric cells was disassembled and the Li metal electrodes were investigated by SEM. For the NL/LE hybrid, a highly uniform NL surface was observed (Fig. 4b), whereas for the NPEM cell, the localized formation of dendrite and mossy Li was observed on the bare Li (Fig. 4c). The inhomogeneous Li deposition implies that the Li deposition/dissolution reaction is concentrated at sites with lower resistance levels to lower the large polarization. These results emphasize the importance of attaining low polarization for stable Li deposition/dissolution and support the benefits of the NL/LE hybrid approach. The destabilized interfaced for the NPEM was further supported by the results of an impedance analysis of the Li/Li symmetric cells, as shown in Supplementary Fig. S5. According to the impedance levels after five cycles, the bulk resistance (R_b_), which corresponds to the high-frequency intercept on the Z’ axis, was 873.1 Ω cm^2^ for the NPEM cell, which is much higher than the corresponding values for the LE (14.2 Ω cm^2^) and NL/LE (25 Ω cm^2^) cells. The interfacial resistance (R_int_), which is generally regarded as the sum of the resistance of the SEI layer and the charge transfer resistance, was determined from the diameter of the lower frequency semi-circle (100 Hz). The R_int_ value of the Li/NPEM cell was around 598.5 Ω cm^2^ after the fifth cycle (50 h), which is considerably larger than those of the LE cell (209.4 Ω cm^2^) and the NL/LE cell (320.9 Ω cm^2^). This result demonstrates that the large overvoltages for the NPEM cell are attributed not only to the large ohmic resistance of NPEM but also to the destabilized interface triggered by the large ohmic resistance.

**Figure 5:**
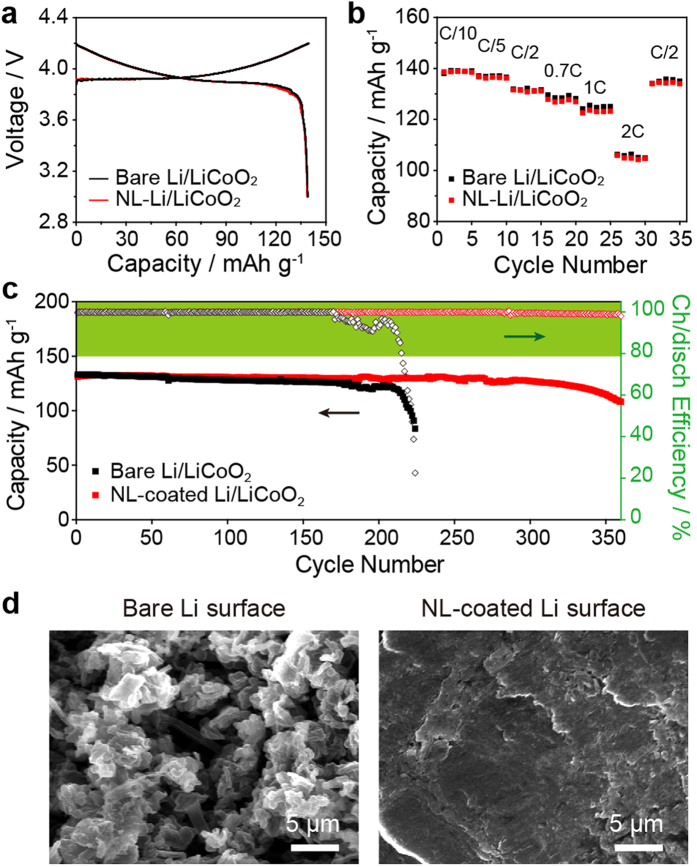
Room-temperature battery performances and SEM analyses of Li/LiCoO_2_ cells employing bare and NL-coated Li electrodes. (**a**) typical charge/discharge curves at a fixed current density of 0.1 C (0.15 mA cm^−2^), (**b**) rate capability at various discharge rates (0.1–2.0 C, 0.15–3.0 mA cm^−2^), (**c**) capacity retention (filled boxes) and coulombic efficiency (empty boxes) with cycling at 0.2 C, and (**d**) Surficial SEM images of the Li metal electrodes after 100 cycles.

To assess the practical applicability of the hybrid approach, a Li/LiCoO_2_ cell employing the NL/LE hybrid (NL/LE hybrid cell) design was tested at room temperature and compared with the LE-based Li/LiCoO_2_ cell (LE cell). It is important to note that LiCoO_2_, which has been successfully commercialized as a LIB cathode material, has excellent cycling stability which is unlimited when combined with anode materials with higher capacities, such as Li metal and Si. Hence, the Li/LiCoO_2_ cell test provides information about the cycling stability of the Li metal electrode. As shown in Fig. 5a, the charge-discharge profiles of the LE and NL/LE hybrid cell were nearly identical. Even at a high discharge rate of 2C (3.0 mA cm^−2^), the discharge capacities of the two cells were nearly indistinguishable, as shown in Fig. 5b. The rate capability data clearly demonstrate the efficacy of the hydride approach in maintaining low cell resistance. The LE cell exhibited gradual capacity fading for 200 cycles and a subsequent abrupt decay of both the discharge capacity and coulombic efficiency, whereas the NL/LE hybrid cell operated stably up to 360 cycles with a loss of only 82.6% capacity from its initial capacity (Fig. 5c). After 100 cycles, the Li/LiCoO_2_ cells were disassembled and the Li metal surfaces were investigated. As shown in Fig. 5d, the degree of surface uniformity of the Li metal electrode was profoundly different between the LE and the NL/NE cell. In the Li/Li symmetric cells, a porous Li layer was formed on the Li metal electrode for the LE cell; however, this type of porous layer was not seen for the NL/NE cell. Furthermore, the C_1s_, O_1s_, and F_1s_ XPS data pertaining to the surface of the NL-coated Li and bare Li examined after five cycles collectively indicated a reduced amount of electrolyte decomposition with the introduction of the NL (Supplementary Fig. S6). Therefore, the homogeneous and stable Li electrodeposition observed for the Li/Li symmetric cell was successfully confirmed with the Li/LiCoO_2_ cells, ensuring the efficacy of this approach.

## Conclusion

In summary, when the NL was introduced onto the Li metal/electrolyte interface coupled with LE, it allowed a uniform distribution of Li^+^ onto the Li metal surface, leading to the homogeneous and stable electrodeposition of the Li metal electrode. Owing to the single-ion conducting capabilities, the low resistance, and the tight adhesion of the NL onto the Li metal, both dendritic Li growth and decomposition of the electrolyte were effectively suppressed. As a result, the Li/Li symmetric cell can operate at a high current density of 10 mA cm^−2^ for more than 2000 h, and the Li/LiCoO_2_ prototype LMB offers cycling stability which lasts for more than 350 cycles. Therefore, the use of a thin NL for a means of Li protection coupled with LE can be regarded as an effective and practical strategy for improving the performance and durability of advanced LMBs based on its effects and its simplicity.

## Methods

### Preparation of NL-coated Li metal electrode

Li metal (Honzo Metal, Japan) foil with a thickness of 150 μm was used as an electrode. A thin NL was formed by spraying a commercial Nafion dispersion onto a FEP (DuPont) film at a properly controlled dose rate. The NL-coated FEP film was then dried at 130 °C in a vacuum chamber for 24 h and subsequently stored in an Ar-filled glove box with a dew point below −90 °C. The stack of the NL-coated FEP film and the Li metal foil was laminated by a roll-pressing machine at room temperature to provide adhesion of the NL onto the Li metal. The FEP film was then detached from the assembly. As a control electrolyte system for NL/LE, a lithiated Nafion 212 membrane (thickness 50 μm, DuPont) as a single-ion polymer electrolyte was used after being swollen with ethylene carbonate (EC)/diethyl carbonate (DEC) (1/1) as a solvent.

### Electrochemical and mechanical characterizations

All reagents used in the experiments were of analytical-grade purity and were used directly without further treatment. A solution of 1 M lithium hexafluorophosphate (LiPF_6_) dissolved in EC/DEC (1/1) was used as a LE. To evaluate the electrochemical performance, CR2032 coin-type cells were fabricated by stacking a polypropylene (PP, Celgard 2400) separator between two electrodes, followed by an injection of electrolyte. A pair of bare or NL-coated Li metal electrodes was used as symmetric cells. For the symmetric cell with the NPEM, only the solvent-swollen Nafion 212 membrane was placed in between two Li metal electrodes; any porous separator was not additionally inserted in the cell. The Li/Li symmetric cells were galvanostatically cycled at a current density of 0.75 mA cm^−2^ for 10 h during each half cycle and at 10 mA cm^−2^, where each half-cycle life lasted for 4 h. For the LiCoO_2_ cathode, slurry consisting of 92 wt% LiCoO_2_ (KD-10, Umicore, Korea), 4 wt% Super-P carbon black (Timcal, Switzerland), and 4 wt% polyvinylidene fluoride (PVdF) binder (Solef® 6020, Solvay Chemicals Inc., USA) dispersed in N-methyl-2-pyrrolidone (NMP, Aldrich) as a solvent was coated onto an Al foil (thickness of 20 μm) and dried in a vacuum chamber at 110 °C for 24 h before use. The mass loading and electrode density were held to 12 mg cm^−2^ and 3.0 g cm^−3^, respectively. The unit cells were cycled between 3.0 and 4.2 V at a current density of 1.5 mA cm^−2^, defined as a 1C rate at room temperature using a battery tester (TOSCAT-3000U, Toyo System). Electrochemical impedance spectroscopy (EIS; Solartron 1255, frequency range from 1 MHz to 0.1 Hz) was measured during charge–discharge cycling at room temperature. To determine the 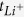 value of the electrolyte, for Swagelok-type Li/Li symmetric cells, potentiostatic polarization experiments were conducted at an applied voltage of 10 mV for 10 h and the impedances (frequency range of 1 MHz-0.1 Hz) were measured before and after the polarization measurement. Indentation-modulus experiments were conducted using a nano-indenter machine (Nano Instruments, USA). For the analysis, the Li-Nafion dispersion was drop-casted onto a silicon wafer and then dried at 110 °C. A partial sample was dipped into an EC/DEC (1/1) solution for 6 h and then dried with filter paper to remove the solvent from the surface.

### Morphological and chemical bonding characterizations

The morphology and bonding characterizations of the electrode surface were carried out via field emission–scanning electron microscopy (FE–SEM; Sirion, FEI), FT–IR (IFS66V/S–HYPERION 3000, Bruker Optik), and XPS (Sigma Probe, Thermo VG Scientific) in a vacuum chamber to prevent Li metal contamination due to moisture and/or oxygen. Before the analysis, each electrode was washed with anhydrous DEC several times in a glove box and dried in a vacuum chamber overnight. The samples were then sealed in laminate pouch bags and transferred into the SEM, FT–IR, and XPS chambers. The FT–IR analysis was carried out in a wave number range of 2,000–900 cm^−1^. All XPS spectra were calibrated against the hydrocarbon peak at a binding energy of 285.0 eV.

## Additional Information

**How to cite this article**: Song, J. *et al.* Ionomer-Liquid Electrolyte Hybrid Ionic Conductor for High Cycling Stability of Lithium Metal Electrodes. *Sci. Rep.* **5**, 14458; doi: 10.1038/srep14458 (2015).

## Supplementary Material

Supplementary Information

## Figures and Tables

**Figure 1:**
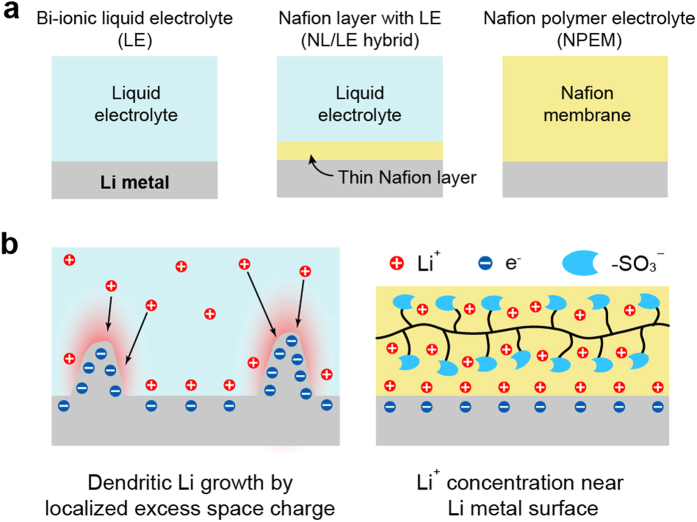
Categorized scheme of the ion conductors. (**a**) configurations of the LE, NL/LE hybrid, and NPEM, (**b**) scheme of the dendritic Li growth resulting from Li^+^ depletion at the interface of LE (left), and for the prevention of dendritic Li due to a uniform Li^+^ concentration at the interface of the Nafion and Li metal for NL/LE and NPEM (right).

## References

[b1] Bruce P. G., Freunberger S. A., Hardwick L. J. & Tarascon, J.-M. Li–O_2_ and Li–S batteries with high energy storage. Nature Mater. 11, 19–29 (2012). 22169914 10.1038/nmat3191

[b2] Lv D. *et al.* Failure Mechanism for Fast-Charged Lithium Metal Batteries with Liquid Electrolytes. Adv. Energy Mater. 5, 1400993 (2014).

[b3] Bates J. B. Oak Ridge, Protective lithium ion conducting ceramic coating for lithium metal anodes and associate method. US Patent 5314765. 1994.

[b4] Wang X., Hou Y., Zhu Y., Wu Y. & Holze R. An Aqueous Rechargeable Lithium Battery Using Coated Li Metal as Anode. Sci. Rep. 3, 1401 (2013). 23466633 10.1038/srep01401 PMC3590562

[b5] Tatsuma T., Taguchi M., Iwaku M., Sotomura T. & Oyama N. Inhibition effects of polyacrylonitrile gel electrolytes on lithium dendrite formation. J. Electroanal. Chem. 472, 142–146 (1999).

[b6] Tatsuma T., Taguchi M. & Oyama N. Inhibition effect of covalently cross-linked gel electrolytes on lithium dendrite formation. Electrochim. Acta 46, 1201–1205 (2001).

[b7] Choi N.-S., Koo B., Yeon J.-T., Lee K. T. & Kim D.-W. Effect of a novel amphipathic ionic liquid on lithium deposition in gel polymer electrolytes. Electrochim. Acta 56, 7249–7255 (2011).

[b8] Aurbach D. Review of selected electrode–solution interactions which determine the performance of Li and Li ion batteries. J. Power Sources 89, 206–218 (2000).

[b9] Gofer Y., Ben-Zion M. & Aurbach D. Solutions of LiAsF_6_ in 1,3-dioxolane for secondary lithium batteries. J. Power Sources 39, 163–178 (1992).

[b10] Naoi K., Mori M., Naruoka Y., Lamanna W. M. & Atanasoski R. The Surface Film Formed on a Lithium Metal Electrode in a New Imide Electrolyte, Lithium Bis(perfluoroethylsulfonylimide) [LiN(C_2_F_5_SO_2_)_2_]. J. Electrochem. Soc. 146, 462–469 (1999).

[b11] Aurbach D. & Zaban A. Impedance spectroscopy of lithium electrodes: Part 1. General behavior in propylene carbonate solutions and the correlation to surface chemistry and cycling efficiency. J. Electroanal. Chem. 348, 155–179 (1993).

[b12] Jeong S.-K. *et al.* Suppression of dendritic lithium formation by using concentrated electrolyte solutions. Electrochem. Commun. 10, 635–638 (2008).

[b13] Suo L., Hu Y., Li H., Armand M. & Chen L. A new class of Solvent-in-Salt electrolyte for high-energy rechargeable metallic lithium batteries. Nat. Commun. 4, 1481 (2013). 23403582 10.1038/ncomms2513

[b14] Mogi R. *et al.* Effects of Some Organic Additives on Lithium Deposition in Propylene Carbonate. J. Electrochem. Soc. 149, A1578–A1583 (2002).

[b15] Lu Y., Tu Z. & Archer L. A. Stable lithium electrodeposition in liquid and nanoporous solid electrolytes. Nature Mater. 13, 961–969 (2014). 25108613 10.1038/nmat4041

[b16] Umeda G. A. *et al.* Protection of lithium metal surfaces using tetraethoxysilane. J. Mater. Chem. 21, 1593–1599 (2011).

[b17] Marchioni F. *et al.* Protection of Lithium Metal Surfaces Using Chlorosilanes. Langmuir 23, 11597–11602 (2007). 17939690 10.1021/la701662r

[b18] Choi N.-S., Lee Y. M., Park J. H. & Park J.-K. Interfacial enhancement between lithium electrode and polymer electrolytes. J. Power Sources 119, 610–616 (2003).

[b19] Belov D. G., Yarmolenko O. V., Peng A. & Efimov O. N. Lithium surface protection by polyacetylene *in situ* polymerization. Synthetic. Met. 156, 745–751 (2006).

[b20] Wu M., Wen Z., Liu Y., Wang X. & Huang L. Electrochemical behaviors of a Li_3_N modified Li metal electrode in secondary lithium batteries. J. Power Sources 196, 8091–8097 (2011).

[b21] Lee D. J. *et al.* Composite protective layer for Li metal anode in high-performance lithium-oxygen batteries. Electrochem. Commun. 40, 45–48 (2014).

[b22] Lee H., Lee D. J., Kim Y.-J., Park J.-K. & Kim H.-T. A simple composite protective layer coating that enhances the cycling stability of lithium metal batteries. J. Power Sources 284, 103–108 (2015).

[b23] Ding F. *et al.* Dendrite-Free Lithium Deposition via Self-Healing Electrostatic Shield Mechanism. J. Am. Chem. Soc. 135, 4450–4456 (2013). 23448508 10.1021/ja312241y

[b24] Zhang Y. *et al.* Dendrite-Free Lithium Deposition with Self-Aligned Nanorod Structure. Nano Lett. 14, 6889–6896 (2014). 25419865 10.1021/nl5039117

[b25] Brissot C., Rosso M., Chazalviel J. N. & Lascaud S. Dendritic growth mechanisms in lithium/polymer cells. J. Power Sources 81, 925–929 (1999).

[b26] Chazalviel J. N. Electrochemical aspects of the generation of ramified metallic electrodeposits. Phys. Rev. A 42, 7355–7367 (1990). 9904050 10.1103/physreva.42.7355

[b27] Tikekar M. D., Archer L. A. & Koch D. L. Stability Analysis of Electrodeposition across a Structured Electrolyte with Immobilized Anions. J. Electrochem. Soc. 161, A847–A855 (2014).

[b28] Schaefer J. L., Yanga D. A. & Archer L. A. High Lithium Transference Number Electrolytes via Creation of 3-dimensional, Charged, Nanoporous Networks from Dense Functionalized Nanoparticles Composites. Chem. Mater. 25, 834–839 (2013).

[b29] Lu Y., Das S. K., Moganty S. S. & Archer L. A. Ionic Liquid-Nanoparticle Hybrid Electrolytes and their Application in Secondary Lithium-Metal Batteries. Adv. Mater. 24, 4430–4435 (2012). 22786760 10.1002/adma.201201953

[b30] Maria S. *et al.* Single-ion BAB triblock copolymers as highly efficient electrolytes for lithium-metal batteries. Nat. Mater. 12, 452–457 (2013). 23542871 10.1038/nmat3602

[b31] Sadoway D. R. *et al.* Self-doped block copolymer electrolyte for solid-state, rechargeable lithium batteries. J. Power Sources 97, 621–623 (2001).

[b32] Ryu S.-W. *et al.* Effect of Counter Ion Placement on Conductivity in Single-Ion Conducting Block Copolymer Electrolytes. J. Electrochem. Soc. 152, A158–A163 (2005).

[b33] Allcock H. R., Welna D. T. & Stone D. A. Synthesis of Pendent Functionalized Cyclotriphosphazene Polyoctenamers: Amphiphilic Lithium Ion Conductive Materials. Macromolecules 38, 10406–10412 (2005).

[b34] Stone D. A., Allcock H. R. & Welna D. T. Lithium-Ion Conductive Polymers as Prospective Membranes for Lithium−Seawater Batteries. Chem. Mater. 18, 4486–4492 (2006).

[b35] Stone D. A., Welna D. T. & Allcock H. R. Synthesis and Characterization of Lithium-Ion Conductive Membranes with Low Water Permeation. Chem. Mater. 19, 2473–2482 (2007).

[b36] Sata T., Sata T. & Yang W. Studies on cation-exchange membranes having permselectivity between cations in electrodialysis. J. Membrane Sci. 206, 31–60 (2002).

[b37] Liang H.-Y., Qiu X.-P., Zhang S.-C., Zhu W.-T. & Chen L.-Q. Study of lithiated Nafion ionomer for lithium batteries. J. Appl. Electrochem. 34, 1211–1214 (2004).

[b38] Wang M., Zhao F. & Dong S. A Single Ionic Conductor Based on Nafion and Its Electrochemical Properties Used As Lithium Polymer Electrolyte. J. Phys. Chem. B 108, 1365–1370 (2004).

[b39] Lu Y. *et al.* Stable Cycling of Lithium Metal Batteries Using High Transference Number Electrolytes. Adv. Energy Mater. 5, 1402073 (2015).

[b40] Surman C. M. (Guilderland), Potyrailo R. A. (Niskayuna) & Morris W. G. (Rexford) US Patent 0320142, 2011.

[b41] Evans J., Vincent C. A. & Bruce P. G. Electrochemical measurement of transference numbers in polymer electrolytes. Polymer 28, 2324–2328 (1987).

[b42] Jin Z., Xie K., Hong X., Hu Z. & Liu X. Application of lithiated Nafion ionomer film as functional separator for lithium sulfur cells. J. Power Sources 218, 163–167 (2012).

[b43] Jin Z., Xie K. & Hong X. Electrochemical performance of lithium/sulfur batteries using perfluorinated ionomer electrolyte with lithium sulfonyl dicyanomethide functional groups as functional separator. RSC Adv. 3, 8889–8898 (2013).

[b44] Xu W. *et al.* Lithium metal anodes for rechargeable batteries. Energy Environ. Sci. 7, 513–537 (2014).

[b45] Monroe C. & Newman J. The Impact of Elastic Deformation on Deposition Kinetics at Lithium/Polymer Interfaces. J. Electrochem. Soc. 152, A396–A404 (2005).

