# Supplementary material for: Ionomer-Liquid Electrolyte Hybrid Ionic Conductor for High Cycling Stability of Lithium Metal Electrodes
Source: Sci Rep. 2015 Sep 28;5:14458. doi: 10.1038/srep14458 (PMC4585981; doi:10.1038/srep14458)
Supplement: Supplementary Information [file srep14458-s1.doc]

**Supplementary Information**

Ionomer-Liquid Electrolyte Hybrid Ionic Conductor for High Cycling Stability of Lithium Metal Electrodes

Jongchan Song, Hongkyung Lee, Min-Ju Choo, Jung-Ki Park*, and Hee-Tak Kim*


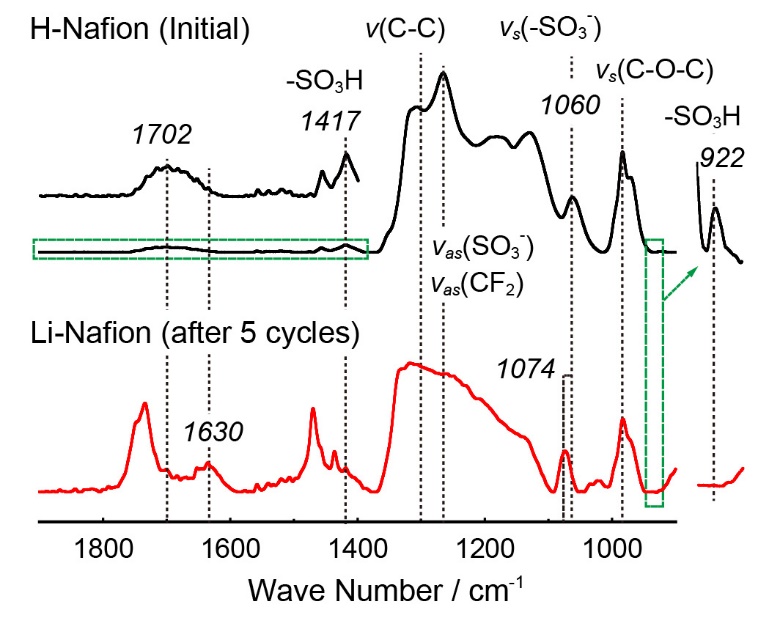


**Supplementary Figure S1.** FT–IR spectra of the Nafion layer on a Li metal electrode before and after charge-discharge galvanostatic cycling, where the cell lasts for five cycles at a current density of 0.75 mA cm^-2^.

During the fabrication of the NL-coated Li electrode, protonated Nafion (H-Nafion), which is more flexible than lithiated Nafion (Li-Nafion), was used for an easier decal transfer process. However, it was transformed to Li-Nafion during galvanostatic cycling, as indicated by a FT
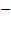
IR analysis (Figure S1). The peak from the SO_3_ symmetric stretch at 1,060 cm^-1^ detected for the NL before cycling was shifted to a slightly higher frequency (1,074 cm^-1^) after five cycles^1,2^. This is ascribed to the stronger interaction with Li^+^ compared to that with H^+ 3^. Also, the S=O stretching (1,417 cm^-1^) and the S-OH stretching (922 cm^-1^) peaks from sulfonic acid (-SO_3_H)^1^ disappeared after the cycling process, confirming the ionic exchange between the proton and the Li^+^. The ion exchange is favorable during the cathodic scan, as the proton reduction potential (2H^+^ + 2e^-^
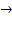
 H_2_) is 3.04 V higher than the Li^+^ reduction potential. Moreover, the absorption peak at 1,702 cm^-1^ disappeared and vibration of the water molecules newly appeared at approximately 1,630 cm^-1^ in the NL spectrum after cycling^4-6^.


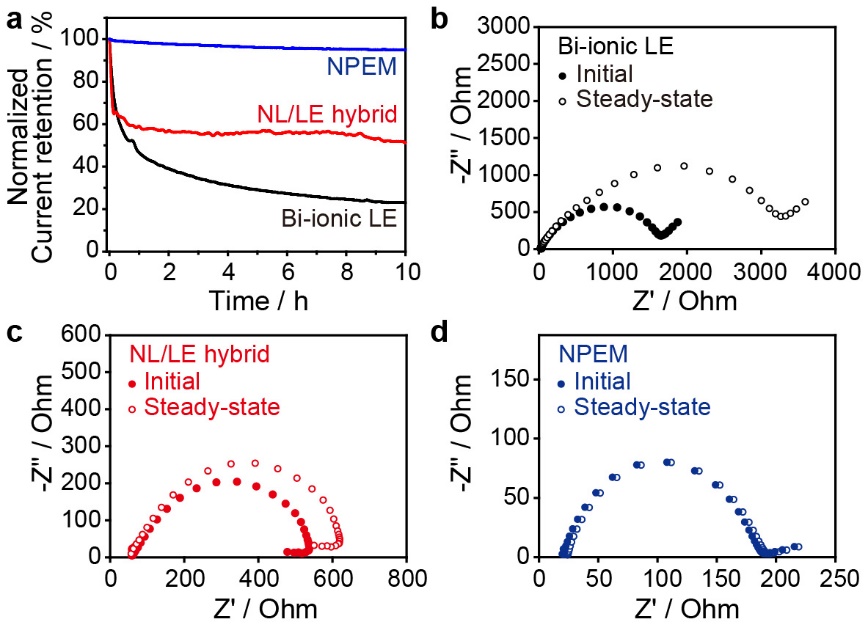


**Supplementary Figure S2.** a) Normalized chronoamperograms, and b-d) Nyquist plots for Li/Li symmetric Swagelok-type cells with bi-ionic LE (1 M LiPF_6_ in EC/DEC (1/1)), NPEM swollen by EC/DEC (1/1), and Li-NL/LE with an applied voltage of 10 mV for 10 h. The frequency range for the impedance measurement was 1MHz to 0.1Hz.

The $t_{Li^{+}}$ values of the LE, NL/LE hybrid, and NPEM were determined using the method by Bruce and Vincent with equation (S1) below on a Swagelok-type cell assembled by sandwiching each electrolyte between two Li metal electrodes.

$t_{Li^{+}}=\frac{I_{SS}\left(\Delta V-I_{0}R_{0}\right)}{I_{0}\left(\Delta V-I_{SS}R_{SS}\right)}$ (S1)

Here, I_0_ and I_SS_ denote the initial and the steady-state current, and R_0_ and R_SS_ are the initial and steady-state interfacial resistance, respectively. △V is the potential difference. The $t_{Li^{+}}$ values obtained from the data in Figure S2 using this equation are summarized in Table S1 below.

**Supplementary Table S1.** Li transference numbers at room temperature for LE, the NL/LE hybrid, and NPEM and measured data from Figure S2.

| Electrolytes | R_0_  [Ω] | R_SS_  [Ω] | I_0_  [A] | I_SS_  [A] | t_Li+_ |
| --- | --- | --- | --- | --- | --- |
| Li/LE | 1645 | 3650 | 39.9 x 10^-6^ | 9.8 x 10^-6^ | 0.451 |
| Li/NPEM | 161 | 165 | 5.2 x 10^-3^ | 4.9 x 10^-3^ | 1.000 |
| Li-NL/LE hybrid | 470.1 | 549.8 | 10.6 x 10^-6^ | 5.8 x 10^-6^ | 0.855 |


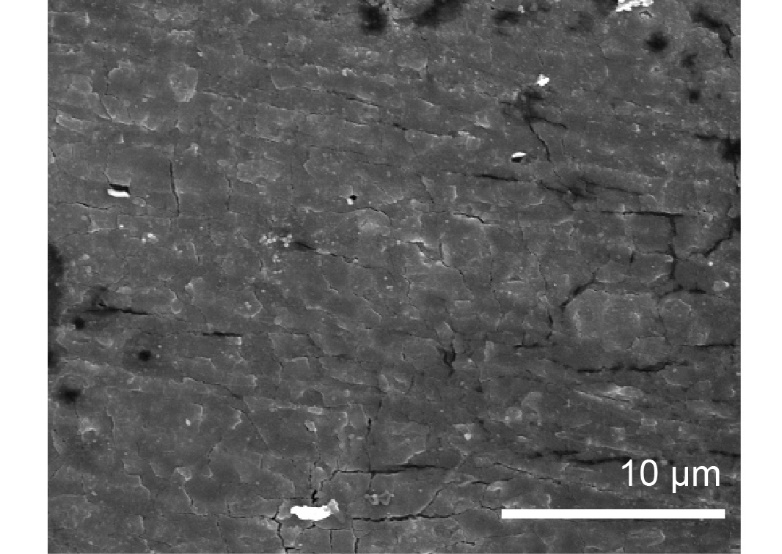


**Supplementary Figure S3.** SEM image of the Li metal surface of a NL-coated Li metal electrode for which the NL was peeled off after 10 cycles at 0.75 mA cm^-2^.


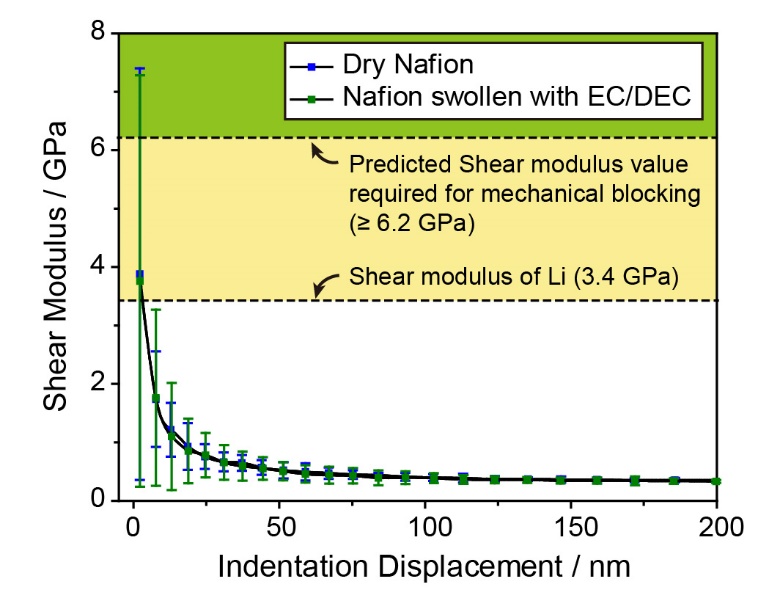


**Supplementary Figure S4.** Comparison of the shear modulus values by means of nano-indentation measurements as a function of the indentation displacement for the Nafion ionomer in a dry state and in a swollen state with EC/DEC (1/1) as a plasticizer.

Mechanical suppression of dendrite growth via a protection layer on a Li metal electrode is a widely used strategy to enhance the cycling stability of Li metal electrodes. According to Monroe’s theoretical model, dendritic Li growth can be mechanically prevented when the shear modulus of the protection layer with a Poisson’s ratio of 0.33 in contact with the Li metal electrode is 1.8 times higher than that of the Li metal. In order to check the mechanical suppression effect of Nafion, we quantified the shear modulus of a dry NL and a NL swollen with electrolyte using a nano-indentation method. The maximum shear modulus at narrow indentation distance of ~50 nm was 3.88 and 3.76 GPa for the dry and swollen Nafion, respectively. All modulus values for the NLs decreased as the indentation depth increased, with the dry and swollen Nafion finally becoming saturated at an indentation displacement of 200 nm at 0.35 and 0.33 GPa, respectively. These values are lower than the critical shear modulus (G’ > 6.2 GPa) required to suppress dendrite growth, as calculated by Monroe and Newman based on linear elasticity theory, indicating that the stabilizing effect of the NL is more likely attributed to its single-ion conducting characteristic.


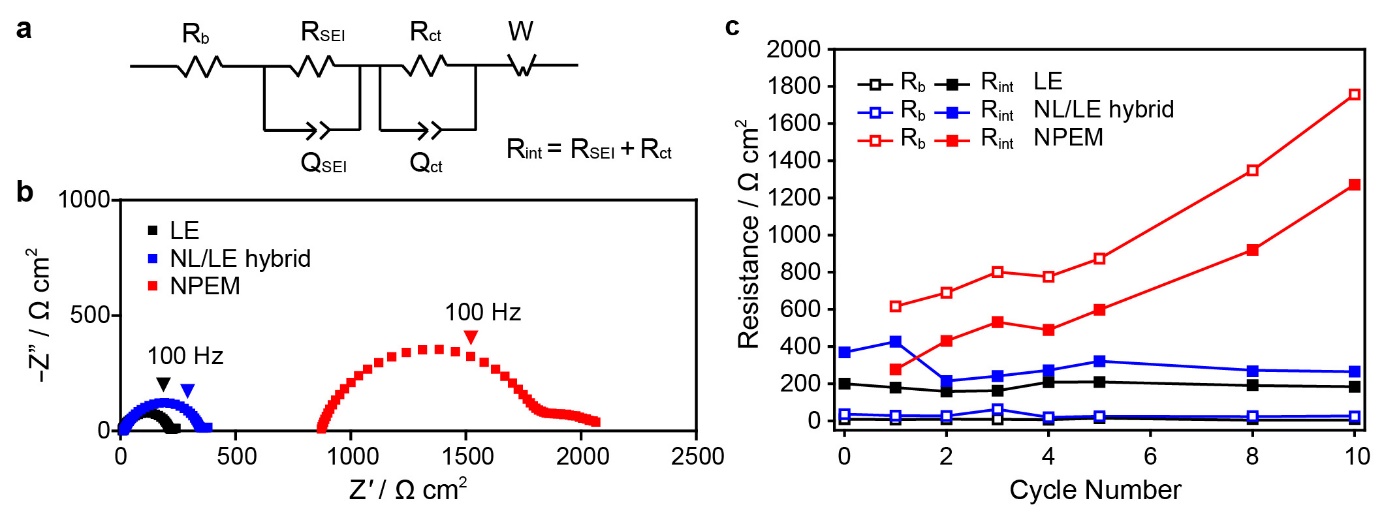


**Supplementary Figure S5.** EIS analysis of Li/Li symmetric cells depending on the electrolyte configuration at current densities of 0.08 mA cm^-2^ for the initial five cycles and 0.1 mA cm^-2^ for the next five cycles. Each cycle lifetime lasts 10 h. a) Equivalent circuit model used for the Nyquist plot fitting. b) Nyquist plots of the impedances for the LE, NL/LE, and NPEM cells after the initial five cycles. c) Bulk (R_b_) and interfacial resistances (R_int_) after repeated cycling.

An EIS analysis provides a more quantitative description of the Li/electrolyte interfaces. The impedance data was described with a circuit model (Figure S5a) which features the bulk resistance (R_b_), interfacial resistance (R_int_), interfacial capacitance of the constant phase element (Q_int_), and the Warburg diffusion element (*W*). In order to reflect the inhomogeneity of the Li electrode interface, CPE is used instead of the ideal capacitance. By fitting the circuit model to the data, these circuit model parameters were determined and plotted as a function of the number of cycles, as shown in Figure S4c. The NPEM cell is clearly differentiated from the other cells in that both R_b_ and R_int_ increased with the number of cycles. In detail, the R_int_ ratio between the fifth and the first cycle was 1.17, 0.75, and 2.16 for the LE, the NL/LE, and the NPEM cell, respectively, while the R_b_ ratio between the fifth and the first cycle was 1.85, 0.89, and 1.42 for the LE, NL/LE, and NPEM cell, respectively.


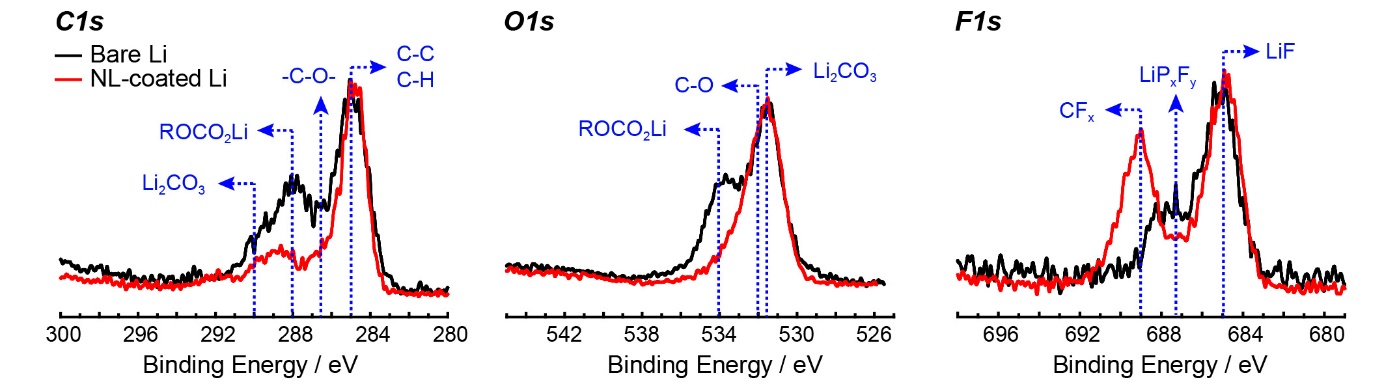


**Supplementary Figure S6.** XPS spectra of the surface of a bare and a NL-coated Li metal anode in Li/LiCoO_2_ cells after the fifth cycle

Considering the characteristics of the NL/LE hybrid, the effect of the NL on the prevention of electrolyte decomposition works in two ways: i) the suppression of the ramified Li deposition reduces the Li surface on which electrolyte decomposition occurs, and ii) the tightly adhered NL can prevent any direct access of the bulk LE to the Li metal electrode and any consequent chemical reaction between the LE and Li. Considering the influence of the NL/LE hybrid on the electrolyte decomposition process at the Li metal electrode, the chemical structures of the Li interfaces were investigated by XPS. Figure S6 compares the C1s, O1s, and F1s spectra of fully charged LE and NL/LE hybrid-based Li/LiCoO_2_ cells after five cycles. In the C1s spectra, the NL/LE hybrid cell exhibited pronounced suppression of the peaks at 286.8, 288, and 290 eV compared with the LE cell; these peaks originate from the -C-O- species, Li-alkyl carbonate (ROCO_2_Li), and Li-carbonate (Li_2_CO_3_), respectively^8,9^. In the O1s spectra, the peak at 534 eV from ROCO_2_Li^10^, evident for the LE cell, was not observed for the NL/LE hybrid cell. The peaks from Li-alkyl carbonate and Li-carbonate are decomposition products from the carbonate solvents of EC and DEC^11^. The F1s spectra of the NL/LE hybrid cell demonstrated the presence of a C-F bond (689 eV) which originates from the perfluorinated ionomer chain^12^. Furthermore, the F1s peak at 687.2 eV from LiPF_6_ salt^13^ did not appear for the NL/LE hybrid cell. The C1s, O1s, and F1s spectra collectively demonstrate that NL effectively prevents LE decomposition, thereby contributing to the enhancement of the electrochemical stability of the Li metal electrode.

**References for Supplementary Information**

1. Buzzoni R., Bordiga S., Ricchiardi G., Spoto G. & Zecchina A. Interaction of H_2_O, CH_3_OH, (CH_3_)_2_O, CH_3_CN, and Pyridine with the Superacid Perfluorosulfonic Membrane Nafion: An IR and Raman Study. *J. Phys. Chem.* **99**, 11937-11951 (1995).

2. Wirguin C. H. Infra-red spectra of perfluorinated cation-exchanged membranes. *Polymer* **20**, 371-374 (1979).

3. Liu N., Li H., Wang Z., Huang X. & L. Chen. Origin of Solid Electrolyte Interphase on Nanosized LiCoO_2_. *Electrochem. Solid St.* **9**, A328-A331 (2006).

4. Falk M. An infrared study of water in perfluorosulfonate (Nafion) membranes. *Can.* *J. Chem.* **58**, 1495-1501 (1980).

5. Quezado S., Kwak J. & Falk M. An infrared study of water–ion interactions in perfluorosulfonate (Nafion) membranes. *Can. J. Chem.* **62**, 958-966 (1984).

6. Ludvigsson M., Lindgren J. & Tegenfeldt J. FTIR study of water in cast Nafion films. *Electrochim. Acta* **45**, 2267-2271 (2000).

7. Monroe C. & Newman J. The Impact of Elastic Deformation on Deposition Kinetics at Lithium/Polymer Interfaces. *J. Electrochem. Soc.* **152**, A396-A404 (2005).

8. Lee H. et al. Chemical aspect of oxygen dissolved in a dimethyl sulfoxide-based electrolyte on lithium metal. *Electrochim. Acta* **123**, 419-425 (2014).

9. Lee H., Lee D. J., Kim Y.-J., Park J.-K. & Kim H.-T. A simple composite protective layer coating that enhances the cycling stability of lithium metal batteries. *J. Power Sources* **284**, 103-108 (2015).

10. Kang S.-H., Abraham D. P., Xiao A. & Lucht B. L. Investigating the solid electrolyte interphase using binder-free graphite electrodes. *J. Power Sources* **175**, 526-532 (2008).

11. Aurbach D. Review of selected electrode–solution interactions which determine the performance of Li and Li ion batteries. *J. Power Sources* **89**, 206-218 (2000).

12. Zhang F.-Y. et al. Quantitative characterization of catalyst layer degradation in PEM fuel cells by X-ray photoelectron spectroscopy. *Electrochim. Acta* **54**, 4025-4030 (2009).

13. Philippe B. et al. Role of the LiPF_6_ Salt for the Long-Term Stability of Silicon Electrodes in Li-Ion Batteries – A Photoelectron Spectroscopy Study. *Chem. Mater.* **25**, 394-404 (2013).
